# On a Certain Form of Hemoptysis Unassociated with Pulmonary Tuberculosis

**Published:** 1866-09

**Authors:** Richard Payne Cotton

**Affiliations:** Physisian to the Hospital for Consumption and Diseases of the Chest, Brompton


### On a certain form of Hemoptysis unassociated with Pulmonary Tuberculosis.

By Dr. Richard Payne Cotton, Physisian to the Hospital for Consumption and Diseases of the Chest, Brompton.

[That hemoptysis is met with in a very considerable number of non-tubercular cases is well known; its occurrence, however, always suggests the presence of tubercle in the lung. There are many conditions which may give rise to hemoptysis.]

In the present instance I am anxious only to draw attention to a not unfrequent, but, so far as I know, little recognized form of non-tubercular hemoptysis, met with chiefly in the female sex, but sometimes also amongst males, generally in the early period of life.

It may simplify my description of this variety of hemoptysis if I give a brief account of two or three cases in point.

A young lady, aged 18, recently arrived from a residence in one of the West India islands, was supposed to be phthisical. I was requested to see her, and report upon the nature of her disease, about which several very conflicting opinions had already been given. She was anæmic, nervous, and out of health; had a dry cough, but had not become thinner; her catamenia were regular, but scanty; her appetite was capricious; and she had had frequent hemoptysis, which there was every reason to believe did not proceed from either the mouth or fauces. The blood, upon examination, was found to be thin and watery, of a dark color, free from coagulum, unassociated with either bronchial or salivary secretion, and in general appearance much resembling a mixture of *red-currant jelly and water.* I was informed that this was its general character. Sometimes it had been considerahle—as much as half a pint in twenty-four hours; at others, it would not exceed a teaspoonful or two during the same period; sometimes it would be scarcely enough to tinge a pocket-handkerchief, and often it would disappear for days together. This state of things had existed for nearly two years, causing great anxiety to the patient and her friends, from a belief that it was indicative of pulmonary disease. Careful examination of the chest, however, failed to elicit any evidence that such was the case. Rest, change of air, and tincture of sesqui-chloride of iron, entirely restored this patient to health. It is now more than three years since I was consulted, and I heard a short time back that the young lady was in perfect health.

A case very similar to this came under my notice two years ago in consultation with Mr. Humpage, of Upper Seymour street. A young lady, aged 24, had long been delicate, and was snpposed by her family to be consumptive. She had become thinner; had had a dry cough for some length of time; and had spat blood. Upon examination, this was found to present an appearance very similar to that described in the preceding case; it looked, in fact, more like watery red-currant jelly than anything else. As in the other patient, there were no decided signs of tubercular disease; and we came to the conclusion that the patient was not phthisical. Time has justified our diagnosis; Mr. Humpage having lately informed me that the young lady had, for some months, lost the symptom which had caused so much alarm, and was in good health.

Another case, which I shall even more briefly relate, came under my notice about eighteen months back. It was that of a young lady, 12 years of age, of slender and somewhat delicate appearance, but free from every symptom of tubercular affection. Mr. J. N. Winter, of Montpelier road, Brighton, frequently saw this patient, and quite agreed with me as to the nature of her disease. She first alarmed her parents by spitting up, just after going to bed one night, a considerable quantity of blood. This was found upon examination to be watery and dark colored—in fact, of the *thin red-currant jelly character* already described. This symptom has recurred at various intervals with more or less intensity, and the child still remains delicate, but without any indications of tubercular disease. At the time of her attacks her tonsils and pharynx are somewhat congested, her gums spongy, and her fingers even have sometimes exuded a little water blood, just as one some times sees in extreme cases of purpura. It is evident that, in this instance, the blood escapes not only from the mucous membrane of the respiratory passages, but also from that of the throat, tonsils and gums.

Other cases of this form of hemoptysis have fallen under my observation; but I shall not specially refer to them in consequence of not knowing their sequel. At least twelve or thirteen have happened in my own wards in the Hospital for Consumption.—Two only of these were males; the rest were females, generally of delicate and nervous appearance, and under the age of 30. Several had very suspicious symptoms of phthisis; but the physical signs failed to exhibit any evidence of pulmonary tuberculosis, and most of them improved in health under appropriate treatment. In every case the expectoration was of the same general character; and sometimes it was mixed more or less with bronchial mucus, slightly tinged perhaps with blood, and sometimes with salivary secretion; but more frequently it was simply watery blood, resembling, as I have described, a mixture of red-currant jelly with water.

The following are the conclusions at which I have arrived from a consideration of the preceding notes:

1st.—There is a form of true hemoptysis in which the expectoration is of a dark color, and of a more or less watery consistence, bearing a close resemblance to a mixture of red-currant jelly and water.

2d.—That such hemoptysis is of non-tubercular origin, and may proceed from any part of the gastro-pulmonary mucous membrane.

3d.—That it is attributable to a morbid and fluid condition of the blood, allied, at least in appearance, to that which is met with in purpura and scurvy.—*Lancet, Dec.* 2, 1865, *p.* 6,18—*Braithwaite's Retrospect.*

